# Supplementary material for: The interactome of intact mitochondria by cross-linking mass spectrometry provides evidence for coexisting respiratory supercomplexes
Source: Mol Cell Proteomics. 2017 Dec 8;17(2):216–32. doi: 10.1074/mcp.RA117.000470 (PMC5795388; doi:10.1074/mcp.RA117.000470)
Supplement: Supplemental Data [file supp_RA117.000470_133922_0_supp_23978_fzffwf.zip › spectra_annotation/mito_spectra_annotation/100-1-7-1-4-1.pdf]

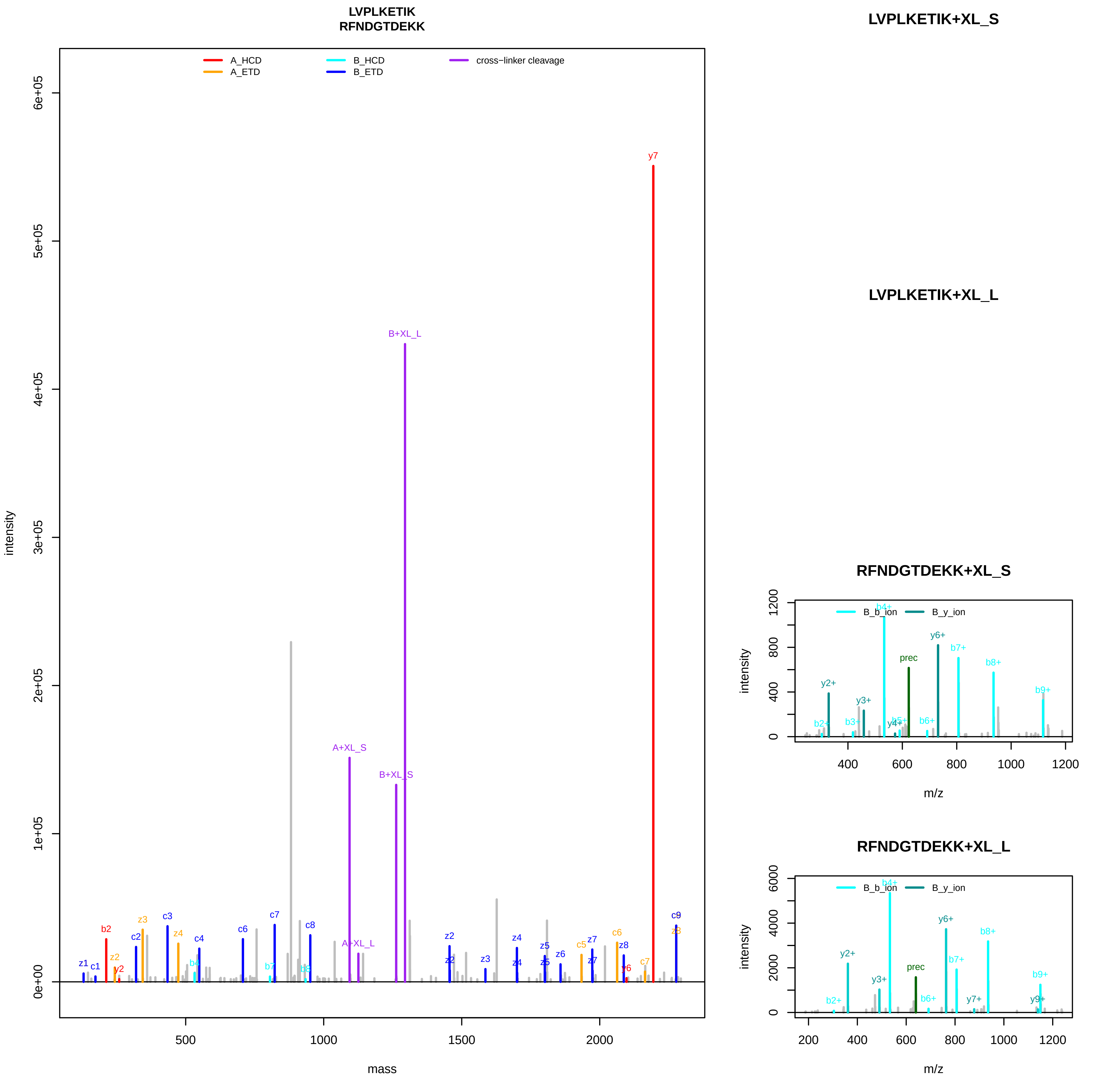
LVPLKETIK
RFNDGTDEKK
A_HCD
A_ETD
B_HCD
B_ETD
cross−linker cleavage
intensity
mass
0e+00
1e+05
2e+05
3e+05
4e+05
5e+05
6e+05
500
1000
1500
2000
y7
B+XL_L
A+XL_S
B+XL_S
A+XL_L
b2
y2
z2
z3
c2
c3
z4
c4
b4
c6
c7
b7
b8
c8
z1
c1
z2
z3
z4
z5
z6
z7
c5
c6
z8
y6
c7
c9
z3
LVPLKETIK+XL_S
LVPLKETIK+XL_L
RFNDGTDEKK+XL_S
B_b_ion
B_y_ion
intensity
m/z
0
400
800
1200
400
600
800
1000
1200
b4+
y6+
b7+
prec
b8+
b9+
y2+
y3+
b2+
b3+
y4+
b5+
b6+
RFNDGTDEKK+XL_L
B_b_ion
B_y_ion
intensity
m/z
0
2000
4000
6000
200
400
600
800
1000
1200
b4+
y6+
b8+
y2+
b7+
prec
y3+
b2+
b6+
y7+
b9+
y9+
